# Electronic Cigarette Usage Patterns and Perceptions in Adult Australians

**DOI:** 10.3390/toxics11030290

**Published:** 2023-03-21

**Authors:** Alexander N. Larcombe, Emily K. Chivers, Rachel R. Huxley, Arthur (Bill) W. Musk, Peter J. Franklin, Benjamin J. Mullins

**Affiliations:** 1 Respiratory Environmental Health, Wal-yan Respiratory Research Centre, Telethon Kids Institute, Nedlands, WA 6009, Australia; 2 Occupation, Environment and Safety, School of Population Health, Curtin University, Perth, WA 6845, Australia; 3 The George Institute for Global Health, University of New South Wales, Newtown, NSW 2042, Australia; 4 Faculty of Health, Deakin University, Burwood, VIC 3125, Australia; 5 School of Population and Global Health, University of Western Australia, Crawley, WA 6009, Australia; 6 Department of Respiratory Medicine, Sir Charles Gairdner Hospital, Nedlands, WA 6009, Australia

**Keywords:** electronic cigarette, usage patterns, perceptions, Australian adults

## Abstract

Despite their increasing popularity, and Australia’s unique regulatory environment, how and why Australian adults use e-cigarettes and their perceptions of their safety, efficacy and regulation have not been extensively reported before. In this study, we screened 2217 adult Australians with the aim of assessing these questions in a sample of current or former e-cigarette users. A total of 505 out of 2217 respondents were current or former e-cigarette users, with only these respondents completing the full survey. Key findings of this survey included the high proportion of respondents who indicated they were currently using e-cigarettes (307 out of 2217 = 13.8%), and the high proportion of current e-cigarette users that were also smokers (74.6%). The majority of respondents used e-liquids containing nicotine (70.3%), despite it being illegal in Australia without a prescription, and the majority bought their devices and liquids in Australia (65.7%). Respondents reported using e-cigarettes in a variety of places, including inside the home, inside public places (where it is illegal to smoke tobacco cigarettes), and around other people—which has implications for second and third hand exposures. A significant proportion of current e-cigarette users (30.6%) thought that e-cigarettes were completely safe to use long-term, although in general, there was a large amount of uncertainty/ambivalence with respect to perceptions of e-cigarette safety and efficacy as smoking cessation tools. This study shows that e-cigarette use is common in Australia, and that appropriate dissemination of unbiased research findings on their safety and efficacy in smoking cessation is urgently required.

## 1. Introduction

Despite their increasing popularity in many countries, [1,2] including Australia [3], there is a paucity of recent information detailing the usage patterns, and perceptions about electronic cigarettes (e-cigarettes) in Australian adults [4,5]. This is important due to the significantly increased uptake of e-cigarettes in Australians aged over 60 years between 2016 and 2019 (2.4 fold increase for 60- to 69-year-olds, and 3.1 fold increase for those aged over 70 years) [3], and the knowledge that older adults are increasingly turning to e-cigarettes as a potential way to quit or reduce their tobacco smoking [6]. In Australia, current use of e-cigarettes (defined by use daily, weekly, monthly, or less than monthly) more than doubled in all adults aged over 18 between 2016 and 2019 (1.2 to 2.6%) [3]. Further, the legal and regulatory status of e-cigarettes and related devices in Australia is complex, and more restrictive than many other countries [7] which means that data generated in other countries cannot be easily generalised to Australia [8]. In Australia, e-cigarette regulation is shared across Commonwealth and State/Territory governments, and they are covered by multiple pieces of legislation [7,9]. Some Australian jurisdictions ban the sale of e-cigarettes and their components entirely [10]. The purchase of e-liquids or e-cigarette devices containing nicotine is illegal without a prescription throughout Australia. There are further restrictions on what e-liquids with nicotine can contain (with a small list of banned ingredients) [11].

To date, some studies have investigated aspects of e-cigarette awareness, usage patterns and perceptions in an Australian context. These studies often focus on a specific demographic such as socioeconomically disadvantaged smokers [12], Aboriginal and Torres Strait Islanders [13], illicit drug users [14] or adolescents [15,16,17,18]. This attention is clearly warranted, as concerns around harm minimalization and renormalisation of tobacco smoking emerge [19]. More recent Australian studies often focus on awareness and usage rates of e-cigarettes, and perceptions surrounding their use. For example, using data obtained from the 2016 National Drug Strategy Household Survey [NDSHS], Chan et al. (2019) estimated that 1.2% of Australian adults were current e-cigarette users, and 0.5% used them daily [8]. They found positive associations between e-cigarette use and being male, young, a smoker and levels of psychological distress. These results were similar to those previously found using a telephone-based survey in New South Wales [20]. Jongenelis and colleagues recently published a series of studies investigating perceptions of e-cigarettes in 18–25-year-old Australians, with foci on associations between e-cigarette use and smoking, and regulation [15,16,17,18]. They reported a lack of understanding of the potential harms of e-cigarette usage, and a belief that e-cigarettes are effective smoking cessation tools.

The aforementioned studies provide a wealth of valuable information regarding e-cigarette use by Australians, although specific details on key aspects of e-cigarette use, such as what types of devices and e-liquids adult Australian “vapers” are using, how/where they are using them, how/where they obtain their supplies, and their perceptions of e-cigarette safety and efficacy as tobacco smoking cessation tools etc are largely missing [21]. In this study, we aimed to try to fill this knowledge gap by conducting an online national population survey of Australian adults. Our goals were twofold: firstly to acquire a range of knowledge about e-cigarette use in Australian adults, and secondly, to build-upon and update existing literature in this area. Ultimately, these data will improve our understanding of the Australian e-cigarette environment, which is vital in allowing researchers, clinicians, and policy makers to make informed decisions and to better understand what types of e-cigarettes Australian adults are using, where they are obtaining them and how/why they are using them.

## 2. Materials and Methods

### 2.1. Procedures

An online survey was conducted in March 2020 to obtain data on e-cigarette usage in Australian adults (aged > 18 years). Respondents were drawn from a panel of ~113,000 participants held by an online survey provider (Octopus Group). Approximately 18,000 participants were randomly notified of the survey availability via the survey app, email or by logging into the website. The survey initially consisted of two screening questions that filtered out potential participants if (i) they had never used an e-cigarette or other vaping device and/or (ii) they, or anyone in their family, worked in the tobacco, e-cigarette, or nicotine replacement product industries. All responses were anonymous, and respondents received a small financial incentive for participation. The study was approved by the Curtin University Human Research Ethics Committee (HRE2020-0072).

### 2.2. Survey Questions

Participants who were not filtered out by the screening questions were presented with a series of 39 questions to assess e-cigarette usage patterns, practices, and perceptions (Supplementary Methods). These questions were designed such that respondents could complete the survey within 10 to 15 min. These questions were divided into three main themes: (i) demographics and cigarette smoking status, (ii) e-cigarette use, and (iii) experiences and perceptions about e-cigarettes.

### 2.3. Data Analyses

Respondents were divided into “current” (used e-cigarettes in the past 30 days), or former (used e-cigarettes in the past, but not in the last 30 days) users; therefore, “current” and “former” refer to e-cigarette users (and not tobacco smokers) throughout. In some cases we divided respondents into age categories: 18–25 (n = 94), 26–39 (n = 212) and 40+ years (n = 199) as different ages may have different motivations for e-cigarette usage [22,23]. Descriptive statistics were calculated, and differences between current and former users, and the different age categories were assessed using Chi-square (χ^2^) analyses with the adjusted residuals post-hoc technique with Bonferroni adjustment [24]. Chi-square analyses were conducted using Microsoft Excel for Office 365 v16. In instances where the assumptions of a χ^2^ test were not satisfied (>20% of samples having n < 5) Fisher’s exact test was used, with FDR (false-discovery rate) adjustment [25]. These analyses were conducted using R statistical software (v3.4.3) [26]. *p*-values of < 0.05 were considered significant.

## 3. Results

### 3.1. Participant Demographics

The survey was started by 2217 potential participants, of which 1712 (77.2%) were excluded via the screening questions, leaving 505 participants (current users; n = 307 and former users; n = 198; Table 1). Respondents were most likely to be male (61.0%) and employed full-time (51.9%). Almost half (44.3%) had completed, or were undertaking, university studies. Household income was fairly evenly spread across our five categories, with between 17.0% and 22.8% of respondents in each income bracket. Approximately half (47.1%) were 18 to 34 years old, with only 10.4% of current users being ≥55 years old. A fifth (19.6%) of respondents indicated they had asthma.

### 3.2. E-Cigarette Usage and Tobacco Smoking Status

**Figure 1:**
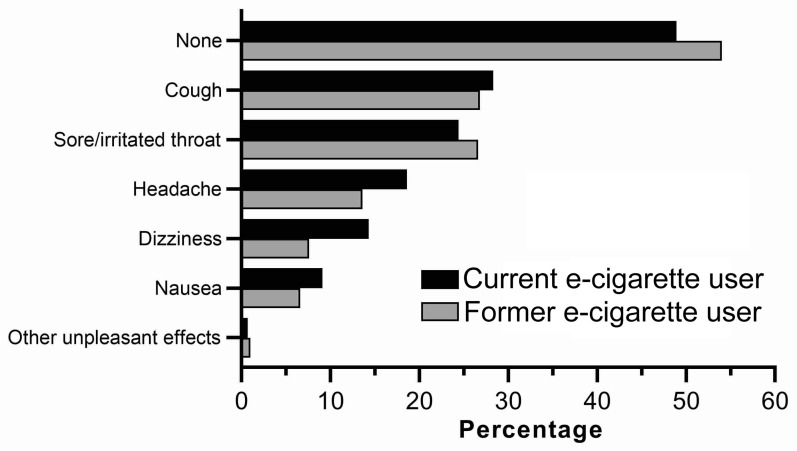
Health effects experienced by Australian adult current and former e-cigarette users. Data are % of respondents—with respondents permitted to select more than one health effect. n = 307 (current e-cigarette users) and n = 198 (former e-cigarette users).

Almost two-thirds of respondents (62.4%) were daily, or occasional tobacco smokers, and 23.2% were former tobacco smokers (Table 2). Current users were more likely to be daily or occasional cigarette smokers (Fisher’s exact test, *p* < 0.002 in both cases). Former users were significantly more likely to respond “I don’t smoke now, but I used to”, “I’ve tried a few times, but never smoked regularly”, or “I’ve never smoked” (Fisher’s exact test, *p* < 0.044 in all cases). Only 1.2% of respondents had never smoked cigarettes. Over half of current users (55.4%) used e-cigarettes at least daily. Most (60.4%) respondents indicated that they experienced cravings to use e-cigarettes “sometimes” or “frequently” (Table 2). E-cigarettes were most commonly used “as needed throughout the day with no defined sessions” (31.1%) or “in separate sessions, similar to smoking a cigarette” (31.3%). The most common place for use was outside respondents’ homes (70.9%). Most respondents (66.9%) indicated that they typically could not use e-cigarettes in places cigarette smoking is banned. Approximately half (49.1%) had experienced unpleasant health effects, with cough (27.7%) and sore/irritated throat (25.1%) being the most common (Figure 1).

**Figure 2:**
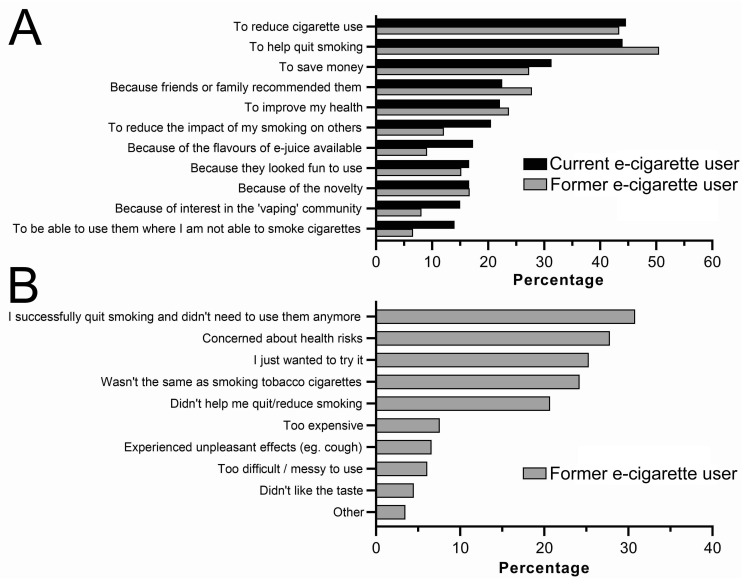
Reasons Australian adult current and former e-cigarette users commenced (**A**) or stopped (**B**) e-cigarette use. Data are % of respondents—with respondents permitted to select more than one reason. n = 307 (current e-cigarette users) and n = 198 (former e-cigarette users).

People started and/or stopped using e-cigarettes for a range of reasons (Figure 2). Of the respondents who stated they started using e-cigarettes “to help quit smoking”, 18.7% also stated they stopped e-cigarette use because they successfully quit tobacco smoking. A similar proportion (13.5%) stated that they stopped using e-cigarettes because they did not help them quit or reduce tobacco smoking. Of the respondents who stated that they do not smoke tobacco cigarettes now, but used to, 61.5% stated they started using e-cigarettes to help quit tobacco smoking. Of these, 35.9% also stated that they stopped using e-cigarettes because they successfully quit tobacco smoking, while only 8.5% stated they stopped using e-cigarettes because they did not help them quit tobacco smoking.

### 3.3. Devices and Liquids Used

Most (72.9%) respondents used a vape-pen or tank/vape-mod device (Supplementary Table S1) as their main device. Current users were significantly more likely to use a sub-ohm device (χ^2^ = (2, n = 505) = 68.75, *p* < 0.001) and were more likely to adjust e-cigarette settings than former users (χ^2^ = (2, n = 505) = 30.59, *p* < 0.001). The most popular sources of devices and e-liquids were Australian physical stores (47.5%) and online suppliers (Australian = 18.2%, overseas = 18.0%). Of the respondents who knew their preferred nicotine level, 78% used e-liquids containing nicotine (Supplementary Table S1). The most popular flavours were tobacco (33.7%), fruit (30.5%) and menthol/mint (19.8%). Un-flavoured e-liquid use was rare (2.6%). Most (50.1%) respondents did not know/did not care about the excipient ratio of their e-liquids; however, current users were significantly more likely to prefer either 50:50 or “High PG” e-liquids (χ^2^ = (4, n = 505) = 48.91, *p* < 0.001).

### 3.4. Perceptions of Safety and Efficacy

For most questions in this section, a substantial proportion of survey participants chose “neutral” as their response (Table 3). We offered this as it allowed an option for respondents not wishing to select a preference [27], although it also provides an “easy-out” and permits respondents to avoid thinking about the question [28]. Current users, and respondents over 40 years of age, generally perceived e-cigarettes to be safer compared with former, and younger users (Table 3).

Most respondents agreed/strongly agreed that (i) e-cigarettes are useful in helping people quit or reduce smoking (67.7%), (ii) that people who smoke tobacco cigarettes should switch to e-cigarettes to improve their health (51.3%), and (iii) people who have never smoked tobacco cigarettes should not use e-cigarettes (50.5%; Table 3).

### 3.5. Perceptions of E-Cigarette Regulation

Between 25.9% and 30.9% of respondents answered “neutral” for questions focussed on e-cigarette regulation (Table 4). Almost half (47.3%) agreed/strongly agreed that e-cigarettes should be regulated in the same way as tobacco cigarettes and other tobacco products. Similar proportions agreed/strongly agreed (36.0%) or disagreed/strongly disagreed (34.7%) that the government should allow e-cigarettes to be used in smoke-free areas. Almost half (49.7%) would use a government-approved e-cigarette containing nicotine instead of their current device/liquid (compared with 13.9% who would not).

## 4. Discussion

### 4.1. E-Cigarette Usage and Tobacco Smoking Status

In this cross-sectional online survey of Australian adults who reported being either a current or former e-cigarette user, the greatest proportion of e-cigarette users were young men who were also cigarette smokers. This is consistent with similar surveys conducted in Australia, Europe, and the United States [3,29,30]. Only a small proportion of e-cigarette users in this survey had never smoked (1.2%), which is lower than that reported for adult Australians in the 2019 NDSHS (6.8%) [3]. Additionally, more than half of current users used e-cigarettes at least daily. The NDSHS reported a considerably lower proportion of daily e-cigarette users (9.4%), which possibly reflects differences in sampling. We acknowledge that our study population is a self-selected group which is not directly comparable to the NDSHS. A potentially important finding of the survey was the higher-than-expected proportion of current e-cigarette users that had doctor diagnosed asthma (19.4%). This is considerably higher than the 11% prevalence for the Australian population [31]. Numerous studies have reported associations between e-cigarette use and asthma prevalence [32,33,34], suggesting that more research is required to better understand the long-term health impacts of e-cigarette use.

### 4.2. Initiation and Cessation of E-Cigarette Usage

The main reasons for initiating e-cigarette use were for smoking cessation, to save money, because they were recommended to the user and for perceived health benefits. Similar reasons were cited for e-cigarette initiation in previous studies [35,36]. This indicates a perception that e-cigarettes are effective smoking cessation tools [37] and healthier alternatives to smoking [18,38]. Recent meta-analyses on the efficacy of e-cigarette use in smoking cessation have provided inconclusive results [39,40]; however, a recent Cochrane review found there is “high-certainty evidence that ECs with nicotine increase quit rates compared to NRT and moderate-certainty evidence that they increase quit rates compared to ECs without nicotine” [41]. There are also data which suggest that tobacco smokers may substitute all or some of their cigarettes with e-cigarettes [42], supporting the perception by users that they are useful smoking cessation tools. Whether they are or are not effective in this capacity is still open to debate, and beyond the scope of this study [40].

The most popular reason for stopping e-cigarette use was that the user successfully quit smoking and did not need e-cigarettes anymore, indicating that some respondents successfully used e-cigarettes to help them quit smoking. To the best of our knowledge, a similar finding has not been previously reported. More commonly, e-cigarette users report stopping use because they were not the same as smoking cigarettes, or that they did not help them quit/reduce smoking [35]. These contradictory findings further add to the uncertainty of the efficacy of e-cigarettes in smoking cessation [39,40].

### 4.3. Devices and Liquids Used

Vape-pen or tank/vape mod type devices were the most popular, which is likely due to the relative ease of buying these types of devices from Australian brick-and-mortar stores. A 2018 systematic review of e-cigarette type preferences showed similar results [43]; however, all reviewed studies were published before 2017—prior to cartridge-based systems and disposables becoming popular. Contemporary data show that cartridge-based systems usage has recently increased significantly [44], and while tank/vape mod systems are still popular [45], their popularity is declining [44].

Most respondents used e-liquids containing nicotine even though at the time the survey was conducted, it was illegal to use, sell or buy nicotine for use in e-cigarettes in Australia without a prescription/permit. Only 14.7% of current users stated that their preferred nicotine concentration was 0 mg/mL (Supplementary Table S1). Previous research shows that nicotine-free e-liquids are rarely used, with levels between ~6 and 18 mg/mL being most popular [43]. This is of concern as there are known health impacts associated with nicotine, including cardiovascular effects [46], impacts on neurodevelopment [47], and risks for poisoning [48]. Tobacco, fruit and menthol/mint were the most common flavours used, which is the same order of preference reported for adults from 33 countries [49]. A survey-based study of young-adult (18–25 years) Australians showed fruit flavours to be popular (55%); however, tobacco and mint/menthol (17% combined) were not [50]. This is likely due to the smoking status and different age groups studied [43]. These findings have important implications as certain flavouring chemicals are associated with adverse health outcomes [51], and that the availability of a range of flavours is associated with e-cigarette use initiation and continuation [52].

Most respondents did not know their preferred excipient, while current users showed a slight preference for either 50:50 PG:VG or high-PG e-liquid. Higher PG levels produce more of a “throat-hit”—which may be preferred by current or former cigarette smokers, although the PG:VG ratio has minimal impact on smokers preferences for e-liquids [53]. Excipient preferences are rarely reported in the literature; however, one early study showed users had a clear preference for high VG e-liquids [54]. This was not apparent in our data, which may be a reflection of the high proportion of tobacco smokers in our sample.

### 4.4. Perceptions of Safety and Efficacy

E-cigarettes were generally perceived as safer than tobacco cigarettes, and that they are effective smoking cessation tools. Current and older users often viewed e-cigarettes more positively than former, or younger users. Many responses to questions about e-cigarette safety were also answered as either “neutral” or “don’t know” which reinforces the idea that users are uncertain (or do not care) about the relative safety of e-cigarettes, and that clear and appropriate dissemination of unbiased research findings is required.

Previous studies on e-cigarette perceptions in Australians have largely focussed on adolescents and young adults. For example, Jongenelis and colleagues recently reported a lack of understanding of the potential harms of e-cigarette usage, but a belief that e-cigarettes are effective smoking cessation tools [15,16,17,18]. In surveys conducted in 2017 and 2018, Wamamili and colleagues found that 75.0% of Australian university students ≤ 25 years old perceived e-cigarettes to be less harmful than tobacco cigarettes, with this number increasing to 100% in “exclusive vapers” [55]. Young adult Australians have been shown to be largely unaware of the health risks associated with e-cigarettes, and misinformed about their efficacy as smoking cessation tools [56].

We identified differences in safety perceptions between current and former users, but fewer differences based on age. Current users generally perceived e-cigarettes as less harmful than tobacco cigarettes [57]. Such perceptions can be based on a lack of available information [58]. In our study, older adults (40+ years) were more than twice as likely to disagree that e-cigarettes are just as harmful, or more harmful than tobacco cigarettes compared with younger adults. They were also almost twice as likely to strongly agree that e-cigarettes should not be used by people who have never smoked. These responses imply that older Australian e-cigarette users believe e-cigarettes should only be used by tobacco smokers, preferentially in a smoking cessation context. We also found that younger adults (18–25 years) were almost three times more likely to disagree with the statement that “people who have never smoked should not use e-cigarettes” compared with older-adults. This agrees with previous literature which shows that adolescents and young-adults do not view e-cigarettes as smoking cessation or harm reduction tools, but as relatively harmless devices associated with fun, flavour experimentation, and discreet use of nicotine [59,60].

A considerable proportion of respondents (37.6%) considered e-cigarettes to be safe around other people (Table 3) or thought they should be allowed in smoke-free areas (36.0%). This may be due to a lack of awareness of the harmful components of second-hand e-cigarette aerosols [61], and again suggests that appropriate dissemination such information is required [62].

Most respondents (67.7%) believed that e-cigarettes are helpful in helping people quit or reduce smoking. Surprisingly, there is a paucity of recently published data exploring this in adult populations, with key studies being relatively old [63], or focused on adolescents [18].

### 4.5. Regulation

There were few strong opinions with respect to e-cigarette regulation (Table 4). A lower proportion of respondents (47.3%) were in favour of regulating e-cigarettes in the same way as tobacco cigarettes than reported in some previous studies [16,64,65]. This difference is likely due to our sample population all being current or former e-cigarette users and adults, as opposed to the general population and/or adolescents. It might also be partially due to the regulatory environment in Australia whereby Australian e-cigarette users may see greater regulation being an avenue to improve access to e-cigarettes. Somewhat surprisingly, over half of current e-cigarette users would use a government-approved e-cigarette and e-liquid instead of their current device/liquid. This, combined with a high degree of uncertainty about e-cigarette safety (Table 4) and the high proportion of users who commenced e-cigarette use to help quit/reduce tobacco smoking suggests that there is an appetite in Australian e-cigarette users for greater clarity and improved safety with respect to their e-cigarette use.

### 4.6. Study Limitations

Outcomes should be interpreted within the context of the study design and its limitations. We acknowledge our survey population size is modest. Further, the data were collected via an online panel, which may not be representative of the Australian adult population, although the respondents were randomly recruited from a web-based panel of over 113,000 potential Australian participants. The study was also cross-sectional in design (meaning we are unable to infer causality) and limited to self-reported data which may be subject to recall bias. Restricting respondents to adults excludes a key demographic of e-cigarette users (adolescents). While self-reports are commonly used in survey-based research, our data were not externally verified.

### 4.7. Conclusions

In conclusion, this study presents a snapshot of data on e-cigarette usage patterns, device/e-liquid preferences and perceptions of their safety and efficacy as cigarette smoking cessation tools in Australia adults in 2020. It shows that e-cigarette use is relatively widespread in Australian adults, despite Australia’s strict regulatory environment, and implies that stronger enforcement of regulations is required. Information on device/e-liquid preferences may be of use in regulatory actions in addition to informing relevant discovery research on the potential for e-cigarettes to impact health (for example choosing appropriate flavours and nicotine levels in in vitro or in vivo toxicological studies). The large amount of uncertainty and ambiguity regarding the safety and efficacy of e-cigarettes as tobacco smoking cessation tools indicates that clear and appropriate dissemination of unbiased research findings is urgently required. With both e-cigarette legislation and the e-cigarette market rapidly changing, further research (and frequently updated data) is needed to more accurately assess e-cigarette usage patterns and perceptions in adult Australians.

## Figures and Tables

**Table 1:** Study participant demographics. Numbers in bold indicate significantly greater than expected compared with current or former user for the same answer.

	Total Sample (n = 505) n (%)	Current E-Cigarette User (n = 307) n (%)	Former E-Cigarette User (n = 198) n (%)	Significance *
**Sex**				
Male	308 (61.0)	**203 (66.1)**	105 (53.0)	*p* = 0.006
Female	194 (38.4)	102 (33.2)	**92 (46.5)**	*p* = 0.006
Nonbinary/prefer not to say	3 (0.6)	2 (0.7)	1 (0.5)	
**Age (years)**				
18–24	80 (15.8)	51 (16.6)	29 (14.6)	
25–34	158 (31.3)	104 (33.9)	54 (27.3)	
35–44	105 (20.8)	65 (21.2)	40 (20.2)	
45–54	96 (19.0)	55 (17.9)	41 (20.7)	
55–65	47 (9.3)	25 (8.1)	22 (11.1)	
Over 65	19 (3.8)	7 (2.3)	12 (6.1)	
**State**				
Australian Capital Territory	6 (1.2)	4 (1.3)	2 (1.0)	
New South Wales	145 (28.7)	97 (31.6)	48 (24.2)	
Northern Territory	1 (0.2)	0 (0.0)	1 (0.5)	
Queensland	113 (22.4)	54 (17.6)	**59 (29.8)**	*p* = 0.012
South Australia	52 (10.3)	34 (11.1)	18 (9.1)	
Tasmania	6 (1.2)	1 (0.3)	5 (2.5)	
Victoria	123 (24.4)	79 (25.7)	44 (22.2)	
Western Australia	59 (11.7)	38 (12.4)	21 (10.6)	
**Employment status**				
Unemployed (seeking work)	44 (8.7)	20 (6.5)	24 (12.1)	
Unemployed (not seeking work)	17 (3.4)	9 (2.9)	8 (4.0)	
Employed (full time)	262 (51.9)	**191 (62.2)**	71 (35.9)	*p* < 0.001
Employed (part time/casual)	114 (22.6)	54 (17.6)	**60 (30.3)**	*p* < 0.001
Student	25 (5.0)	16 (5.2)	9 (4.5)	
Retired	20 (4.0)	8 (2.6)	12 (6.1)	
Unable to work	23 (4.6)	9 (2.9)	14 (7.1)	
**Highest level of education**				
Did not complete high school	54 (10.7)	24 (7.8)	30 (15.2)	
High school certificate	83 (16.4)	48 (15.6)	35 (17.7)	
TAFE/Diploma	14 (28.5)	86 (28.0)	58 (29.3)	
Attended/attending university (but have not graduated)	39 (7.7)	22 (7.2)	17 (8.6)	
Bachelor’s degree	143 (28.3)	98 (31.9)	45 (22.7)	
Completed/completing postgraduate studies	42 (8.3)	29 (9.4)	13 (6.6)	
**Children under 18 at home**				
Yes	234 (46.3)	**165 (53.7)**	69 (34.8)	*p* < 0.001
No	271 (53.7)	142 (43.3)	**129 (65.2)**	*p* < 0.001
**Household income**				
Less than AUD 40,000	86 (17.0)	33 (10.7)	**53 (26.8)**	*p* < 0.001
AUD 40,001 to AUD 70,000	109 (21.6)	62 (20.2)	47 (23.7)	
AUD 70,001 to AUD 90,000	88 (17.4)	61 (19.9)	27 (13.6)	
AUD 90,001 to AUD 130,000	115 (22.8)	79 (25.7)	36 (18.2)	
Above AUD 130,000	94 (18.6)	67 (21.8)	27 (16.6)	
Prefer not to say	13 (2.6)	5 (1.6)	8 (4.0)	
**Aboriginal or Torres Strait Islander**				
Yes	17 (3.4)	13 (4.2)	4 (2.0)	
No	488 (96.6)	294 (95.8)	194 (98.8)	
**Doctor diagnosed respiratory condition #**				
Asthma	99 (19.6)	65 (19.4)	34 (16.4)	
COPD	14 (2.8)	7 (2.1)	7 (3.4)	
Emphysema	11 (2.2)	6 (1.8)	5 (2.4)	
Bronchiectasis	12 (2.4)	10 (3.0)	2 (1.0)	
Chronic bronchitis	17 (3.4)	11 (3.3)	6 (2.9)	
Lung cancer	2 (0.4)	2 (0.6)	0 (0.0)	
Cystic fibrosis	3 (0.6)	3 (0.9)	0 (0.0)	
None of the above	384 (76.0)	231 (69.0)	207 (73.9)	

TAFE = technical and further education, COPD = Chronic obstructive pulmonary disease. * χ^2^ test, except for “Sex”, “State” and “Doctor diagnosed respiratory condition”, which used Fisher’s exact test. # indicates that respondents could select more than one option for that question.

**Table 2:** Smoking history and e-cigarette usage. Numbers in **bold** indicate significantly greater than expected compared with current or former e-cigarette user for the same answer.

	Total Sample (n = 505) n (%)	Current E-Cigarette User (n = 307) n (%)	Former E-Cigarette User (n = 198) n (%)	Significance *
**Smoking Status**				
I smoke daily	198 (39.2)	**138 (45.0)**	60 (30.0)	*p* = 0.002
I smoke occasionally	117 (23.2)	**91 (29.6)**	26 (13.1)	*p* < 0.001
I don’t smoke now, but I used to	117 (23.2)	52 (16.9)	**65 (32.8)**	*p* < 0.001
I’ve tried a few times, but never smoked regularly	65 (12.9)	25 (8.1)	**40 (20.2)**	*p* < 0.001
I’ve never smoked	6 (1.2)	1 (0.3)	**5 (2.5)**	*p* = 0.044
None/don’t know	2 (0.4)	0 (0.0)	2 (1.0)	
**How often do you use e-cigarettes?**				
Constantly throughout the day	63 (12.5)	42 (13.7)	21 (10.6)	
Multiple times per day	151 (29.9)	101 (32.9)	50 (25.3)	
Once per day	33 (6.5)	27 (8.8)	6 (3.0)	
A few times per week	83 (16.4)	61 (19.9)	22 (11.1)	
Weekly	17 (3.4)	10 (3.3)	7 (3.5)	
A few times per month	59 (11.7)	43 (14.0)	16 (8.1)	
Monthly	11 (2.2)	5 (1.6)	6 (3.0)	
Less than monthly	88 (17.4)	18 (5.9)	**70 (35.4)**	*p* < 0.001
**How long have you used e-cigarettes?**				
Less than 6 months	181 (35.8)	79 (25.7)	**102 (51.5)**	*p* < 0.001
6 months to a year	154 (30.5)	104 (33.9)	50 (25.3)	
1 to 2 years	100 (19.8)	**75 (24.4)**	25 (12.6)	*p* < 0.001
More than 2 years	70 (13.9)	49 (16.0)	21 (10.6)	
**Do you experience cravings to use e-cigarettes?**				
Never	187 (37.0)	79 (25.7)	**108 (54.5)**	*p* < 0.001
Sometimes	267 (52.9)	**188 (61.2)**	79 (39.9)	*p* < 0.001
Frequently	38 (7.5)	**35 (11.4)**	3 (1.5)	*p* < 0.001
Don’t know	13 (2.6)	5 (1.6)	8 (4.0)	
**How do you use your e-cigarette?**				
As needed throughout the day with no defined sessions	157 (31.1)	106 (34.5)	51 (25.8)	
In separate sessions, similar to smoking a cigarette	158 (31.3)	101 (32.9)	57 (28.8)	
In social gatherings only	63 (12.5)	25 (8.1)	**38 (19.2)**	*p* = 0.002
Mainly in defined sessions with some ‘top up’ hits as needed	25 (5.0)	15 (4.9)	10 (5.1)	
Occasionally	95 (18.8)	58 (18.9)	37 (18.7)	
Other	7 (1.4)	2 (0.7)	5 (2.5)	
**Where do you use e-cigarettes? #**				
Inside at my home	220 (43.6)	143 (46.6)	77 (38.9)	
Outside at my home	358 (70.9)	229 (74.6)	129 (65.2)	
Inside at public places	74 (14.7)	45 (14.7)	29 (14.6)	
Outside at public places	235 (46.5)	158 (51.5)	77 (38.9)	
Inside at my workplace	26 (5.1)	18 (5.9)	8 (4.0)	
Outside at my workplace	139 (27.5)	95 (30.9)	44 (22.2)	
Alone	170 (33.7)	109 (35.5)	61 (30.8)	
With others present	186 (36.8)	106 (34.5)	80 (40.4)	
In the car	93 (18.4)	64 (20.8)	29 (14.6)	
**Are you typically able to use your e-cigarette in places where smoking is banned?**				
Yes	73 (14.5)	**58 (18.9)**	15 (7.6)	*p* < 0.001
No	338 (66.9)	210 (68.4)	128 (64.6)	
Don’t know	94 (18.6)	39 (12.7)	**55 (27.8)**	*p* < 0.001

* χ^2^ test, except for “Smoking status”, which used Fisher’s exact test. # indicates that respondents could select more than one option for that question.

**Table 3:** Perceptions around e-cigarette safety stratified by current and former e-cigarette users and by age. Data presented are percentages. Numbers in **bold** indicate significantly greater than expected compared within current/former e-cigarette user, or within a particular age category. Sample sizes are: current = 307, former = 198, 18–25 years = 94, 26–39 years = 212, 40+ years = 199.

	Strongly Disagree	Disagree	Neutral	Agree	Strongly Agree	Don’t Know	Significance *
**E-cigarettes are completely safe to use long-term**							
Current	5.2	17.6	37.1	**24.4**	6.2	9.4	*p* = 0.016
Former	**12.1**	**31.8**	33.3	5.1	1.5	16.2	*p = 0.016*
18–25 years	4.3	31.9	31.9	21.3	2.1	8.5	
26–39 years	9.9	21.2	31.1	16.5	6.1	15.1	
40+ years	7.5	21.1	42.2	15.1	3.5	10.6	
**E-cigarettes are significantly less harmful than tobacco cigarettes**							
Current	3.3	7.8	26.1	38.4	**19.2**	5.2	*p* = 0.001
Former	6.1	13.1	32.3	28.8	10.1	9.6	
18–25 years	6.4	7.4	30.9	35.1	13.8	6.4	
26–39 years	3.3	9.0	31.1	33.0	14.2	9.4	
40+ years	4.5	12.1	24.6	36.2	18.1	4.5	
**E-cigarettes are just as harmful as tobacco cigarettes**							
Current	12.1	25.4	28.3	20.5	7.2	6.5	
Former	9.1	20.2	28.3	24.2	8.1	10.1	
18–25 years	7.4	23.4	35.1	23.4	2.1	8.5	
26–39 years	7.1	19.8	28.8	26.9	11.3	6.1	
40+ years	**16.6**	27.1	24.6	16.1	6.0	9.5	*p* = 0.003
**E-cigarettes are more harmful than tobacco cigarettes**							
Current	19.5	27.7	29.6	11.4	5.5	6.2	
Former	16.2	25.3	35.4	9.1	2.5	11.6	
18–25 years	11.7	28.7	35.1	13.8	4.3	6.4	
26–39 years	13.2	24.1	33.5	14.2	6.1	9.0	
40+ years	**26.6**	28.6	28.6	**5.0**	2.5	8.5	*p* = 0.034
**E-cigarettes are safe to use around other people**							
Current	5.2	12.1	34.2	32.9	11.7	3.9	
Former	10.6	15.7	35.9	21.7	5.1	**11.1**	*p* < 0.001
18–25 years	2.1	9.6	40.4	29.8	11.7	6.4	
26–39 years	7.5	17.5	39.2	28.8	9.9	7.1	
40+ years	9.5	11.1	38.2	27.6	7.0	6.5	
**There is sufficient evidence that e-cigarettes are safe**							
Current	5.5	17.9	38.4	**26.7**	**7.8**	3.6	*p* < 0.001
Former	9.6	24.7	39.9	11.6	2.0	**12.1**	*p* < 0.001
18–25 years	3.2	16.0	44.7	23.4	4.3	8.5	
26–39 years	6.6	25.9	33.0	22.2	6.6	5.7	
40+ years	9.5	17.1	42.7	18.1	5.0	7.5	
**People who smoke tobacco cigarettes should switch to e-cigarettes to improve their health**							
Current	3.3	6.8	28.3	44.3	13.4	3.9	
Former	3.5	12.6	35.4	31.8	9.6	7.1	
18–25 years	1.1	11.7	34.0	41.5	8.5	3.2	
26–39 years	3.8	9.4	30.7	38.2	12.7	5.2	
40+ years	4.0	7.5	30.2	39.7	12.6	3.0	
**People who have never smoked tobacco cigarettes should not use e-cigarettes**							
Current	4.6	**18.9**	27.7	27.0	20.2	1.6	*p* = 0.002
Former	3.5	9.1	25.3	29.8	25.8	**6.6**	*p* = 0.002
18–25 years	2.1	**26.6**	30.9	21.3	16.0	3.2	*p* = 0.005
26–39 years	5.7	15.1	27.4	29.7	17.9	4.2	
40+ years	3.5	9.5	24.1	29.6	**30.2**	3.0	*p* = 0.005
**E-cigarettes are useful in helping people quit or reduce smoking**							
Current	2.0	5.5	17.9	49.5	23.8	1.3	
Former	3.5	6.6	23.7	43.4	15.7	**7.1**	*p* = 0.001
18–25 years	1.1	3.2	23.4	46.8	22.3	3.2	
26–39 years	2.8	7.1	19.8	50.9	16.0	3.3	
40+ years	3.0	6.0	19.1	43.2	24.6	4.0	
**I am concerned by recent media reports of e-cigarettes related illnesses and deaths in the United States of America**							
Current	9.1	14.3	8.7	32.6	12.4	2.9	
Former	4.5	9.1	21.7	40.9	14.6	9.1	
18–25 years	9.6	9.6	26.6	34.0	16.0	4.3	
26–39 years	5.2	13.7	26.9	35.8	12.3	6.1	
40+ years	8.5	12.1	24.6	36.7	13.1	5.0	

* χ^2^ test.

**Table 4:** Perceptions around e-cigarette regulations stratified by current and former e-cigarette users and by age. Data presented are percentages. Numbers in **bold** indicate significantly greater than expected compared within current/former e-cigarette user, or within a particular age category. Sample sizes are: current = 307, former = 198, 18–25 years = 94, 26–39 years = 212, 40+ years = 199.

	Strongly Disagree	Disagree	Neutral	Agree	Strongly Agree	Don’t Know	Significance *
**E-cigarettes should be regulated in the same way as traditional cigarettes and other tobacco products**							
Current	8.5	19.2	22.1	34.5	14.3	1.3	
Former	4.5	13.6	31.8	31.3	13.6	5.1	
18–25 years	4.3	14.9	27.7	38.6	11.7	3.2	
26–39 years	4.7	14.2	22.6	36.8	18.9	3.3	
40+ years	10.6	21.1	28.6	27.6	10.1	2.0	
**The government should allow e-cigarettes to be used in smoke-free areas**							
Current	7.2	22.1	27.4	29.6	12.1	1.6	
Former	**16.7**	26.3	26.8	22.2	5.1	3.0	*p* < 0.001
18–25 years	7.4	34.0	23.4	24.5	7.4	3.2	
26–39 years	12.7	20.8	29.2	26.9	8.5	1.9	
40+ years	10.6	22.2	26.8	27.8	10.6	2.0	
**E-cigarettes should be banned until there is more evidence that they are safe**							
Current	15.0	26.4	30.0	16.9	8.8	2.9	
Former	9.6	25.3	32.3	17.2	9.6	6.1	
18–25 years	8.5	28.7	29.8	20.2	6.4	6.4	
26–39 years	10.4	24.1	33.0	18.9	11.3	2.4	
40+ years	17.6	26.6	29.1	13.6	8.0	5.0	
**E-cigarettes should be classified as a drug-based product (like other pharmaceutical nicotine replacement therapies, such as patches and gum)**							
Current	9.1	17.3	29.3	30.0	11.1	3.3	
Former	7.1	16.2	29.3	33.3	9.6	4.5	
18–25 years	7.4	34.0	23.4	24.5	7.4	3.2	
26–39 years	12.7	20.8	29.2	26.9	8.5	1.9	
40+ years	10.6	22.2	26.8	27.8	10.6	2.0	
**I would use a government-approved e-cigarette containing nicotine instead of my current device & juice**							
Current	6.2	7.8	26.4	38.8	17.7	3.6	
Former	6.1	7.6	37.4	29.8	10.1	9.1	
18–25 years	7.4	34.0	23.4	24.5	7.4	3.2	
26–39 years	12.7	20.8	29.2	26.9	8.5	1.9	
40+ years	10.6	22.2	26.8	27.8	10.6	2.0	

* χ^2^ test.

## Data Availability

Not applicable.

## Supplementary Materials

The following supporting information can be downloaded at: https://www.mdpi.com/article/10.3390/toxics11030290/s1, Supplementary Methods, Table S1: Devices, settings, and e-liquid preferences.
