# Supplementary material for: Electronic Cigarette Usage Patterns and Perceptions in Adult Australians
Source: Toxics. 2023 Mar 21;11(3):290. doi: 10.3390/toxics11030290 (PMC10056955; doi:10.3390/toxics11030290)
Supplement: Supplementary file 1 [file toxics-11-00290-s001.zip › Supp Table 1.pdf]

Table S1. Devices, settings and e-liquid preferences. Numbers in bold indicate significantly greater than expected compared with current or former user.

	Total Sample (n=505) n (%)	Current e-cigarette user (n=307) n (%)	Former e-cigarette user (n=198) n (%)	Significance
**What type of device do you use the most?**				
Cig-a-like	85 (16.8)	35 (11.4)	**50 (25.3)**	p < 0.001
Vape-pen	190 (37.6)	114 (37.1)	76 (38.4)	
Tank/vape mod	178 (35.2)	119 (38.8)	59 (29.8)	
JUUL/cartridge type device	52 (10.3)	39 (12.7)	13 (6.6)	
**Is your device "sub-ohm"?**				
Yes	185 (36.6)	**154 (50.2)**	31 (15.7)	p < 0.001
No	44 (8.7)	29 (9.4)	15 (7.6)	
Don't know	276 (54.7)	124 (40.4)	**152 (76.8)**	p < 0.001
**Do you adjust the settings?**				
Yes	229 (45.3)	**169 (55.0)**	60 (30.3)	p < 0.001
No	220 (43.6)	113 (36.8)	**107 (54.0)**	p < 0.001
Don't know	56 (11.1)	25 (8.1)	31 (15.7)	
**Where do you buy your devices and liquids?**				
Physical store in Australia	240 (47.5)	160 (52.1)	80 (40.4)	
Physical store overseas	29 (5.7)	18 (5.9)	11 (5.6)	
Online from an Australian supplier	92 (18.2)	58 (18.9)	34 (17.2)	
Online from an overseas supplier	91 (18.0)	54 (17.6)	37 (18.7)	
I don't know, I didn't buy it	49 (9.7)	16 (5.2)	**33 (16.7)**	p < 0.001
I don't know	4 (0.8)	1 (0.3)	3 (1.5)	
**What is your preferred nicotine level?**				
0mg/mL (no nicotine)	90 (17.8)	45 (14.7)	45 (22.7)	
Less than 6mg/mL (0.6% but not nicotine free)	139 (27.5)	90 (29.3)	49 (24.7)	
7 - 11 mg/mL (0.7 – 1.1%)	130 (25.7)	88 (28.7)	42 (21.2)	
12 – 19 mg/mL (1.2 – 1.9%)	60 (11.9)	42 (13.7)	18 (9.1)	
20 mg/mL or more (over 2%)	26 (5.1)	21 (6.8)	5 (2.5)	
Don't know	60 (11.9)	21 (6.8)	**39 (19.7)**	p < 0.001
**What flavour do you use most?**				
Tobacco	170 (33.7)	102 (33.2)	68 (34.3)	
Fruit	154 (30.5)	105 (34.2)	49 (24.7)	
Menthol/mint	100 (19.8)	53 (17.3)	47 (23.7)	
Candy	29 (5.7)	15 (4.9)	14 (7.1)	
Desert	9 (1.8)	6 (2.0)	3 (1.5)	
Drink/beverage	13 (2.6)	7 (2.3)	6 (3.0)	
Cinnamon	15 (3.0)	11 (3.6)	4 (2.0)	
Un-flavoured	13 (2.6)	7 (2.3)	6 (3.0)	
Other/don't know	2 (0.4)	1 (0.3)	1 (0.5)	
**How many different flavours do you use?**				
1	195 (38.6)	102 (33.2)	**93 (47.0)**	p = 0.003
2 to 3	279 (55.2)	179 (58.3)	100 (50.5)	
4 to 5	24 (4.8)	20 (6.2)	4 (2.0)	
More than 5	7 (1.4)	6 (2.0)	1 (0.5)	
**Preferred PG/VG ratio**				
Don't know	170 33.7)	74 (24.1)	**96 (48.5)**	p < 0.001
Equal	158 (31.3)	**111 (36.2)**	47 (23.7)	p < 0.001
High PG (e.g. 70PG/30VG)	71 (14.1)	**58 (18.9)**	13 (6.6)	p < 0.001
High VG (e.g. 30PG/70VG)	23 (4.6)	20 (6.5)	3 (1.5)	
I don't care about the ratio	83 (16.4)	44 (14.3)	39 (19.7)	

**Do you mix your own e-liquids?**				
Yes	110 (21.8)	**89 (29.0)**	21 (10.6)	$p < 0.001$
No	395 (78.2)	218 (71.0)	**177 (89.4)**	$p < 0.001$
**Do you use nicotine salts?**				
Yes	52 (10.3)	**49 (16.0)**	3 (1.5)	$p < 0.001$
No	404 (80.0)	234 (76.2)	**170 (85.9)**	$p < 0.001$
Don’t know	49 (9.7)	24 (11.8)	25 (12.6)	
