# Supplementary material for: Electronic Cigarette Usage Patterns and Perceptions in Adult Australians
Source: Toxics. 2023 Mar 21;11(3):290. doi: 10.3390/toxics11030290 (PMC10056955; doi:10.3390/toxics11030290)
Supplement: Supplementary file 1 [file toxics-11-00290-s001.zip › Supplementary Methods.pdf]

Supplementary Methods - survey questions

Please tell us a bit about yourself. This information will be used to help us understand differences in e-cigarette use and opinions across different groups of people.

---

1. What is your age? ________________(years)

---

2. What is your gender?

○ Male
○ Female
○ Non-binary
○ Prefer not to say

---

3. What is your postcode? ________________

---

4. What is your employment status?

○ Unemployed (seeking work)
○ Unemployed (not seeking work)
○ Employed (full time)
○ Employed (part time/casual)
○ Student
○ Retired
○ Unable to work

(Please choose option that applies best to you)

---

5. What is your highest level of education?

○ Did not complete high school certificate (less than year 12)
○ High school certificate (year 12)
○ TAFE/Diploma
○ Attending/attended university but have not graduated
○ Graduated university (bachelor's degree)
○ Completing/completed postgraduate studies (masters, PhD etc)

---

6. What is your annual household income? (before tax)

○ Less than $40,000
○ $40,001 - $70,000
○ $70,001 - $90,000
○ $90,001 - $130,000
○ Above $130,000
○ Prefer not to say

(Please select your best estimate if you don't know for sure)

---

7. Do you have children under eighteen living with you?

○ Yes
○ No

---

8. Do you identify as Aboriginal or Torres Strait Islander?

○ Yes
○ No

---

9. Has a doctor ever diagnosed you with:

☐ Asthma
☐ COPD
☐ Emphysema
☐ Bronchiectasis
☐ Chronic bronchitis
☐ Lung cancer
☐ Cystic fibrosis
☐ None of the above

(Please select all that apply)

---

10. What is your tobacco cigarette smoking status?

○ I smoke daily
○ I smoke occasionally
○ I don't smoke now, but I used to
○ I've tried a few times, but never smoked regularly
○ I've never smoked
○ None/don't know

(Please choose option that applies best to you)

---

11. Have you used an e-cigarette in the last 30 days?

○ Yes
○ No

Please tell us how you use your e-cigarette. If you no longer use e-cigarettes, please answer the questions according to your past use.

12. How often do you use e-cigarettes?

○ Constantly throughout the day
○ Multiple times per day
○ Once per day
○ A few times per week
○ Weekly
○ A few times per month
○ Monthly
○ Less than monthly

(If you no longer use e-cigarettes, please answer according to your past use.)

---

13. How long have you used e-cigarettes?

○ Less than 6 months
○ 6 months to 1 year
○ 1 to 2 years
○ More than 2 years

(If you no longer use e-cigarettes, please answer with how long you used them for before stopping)

Please use this image to answer the question below

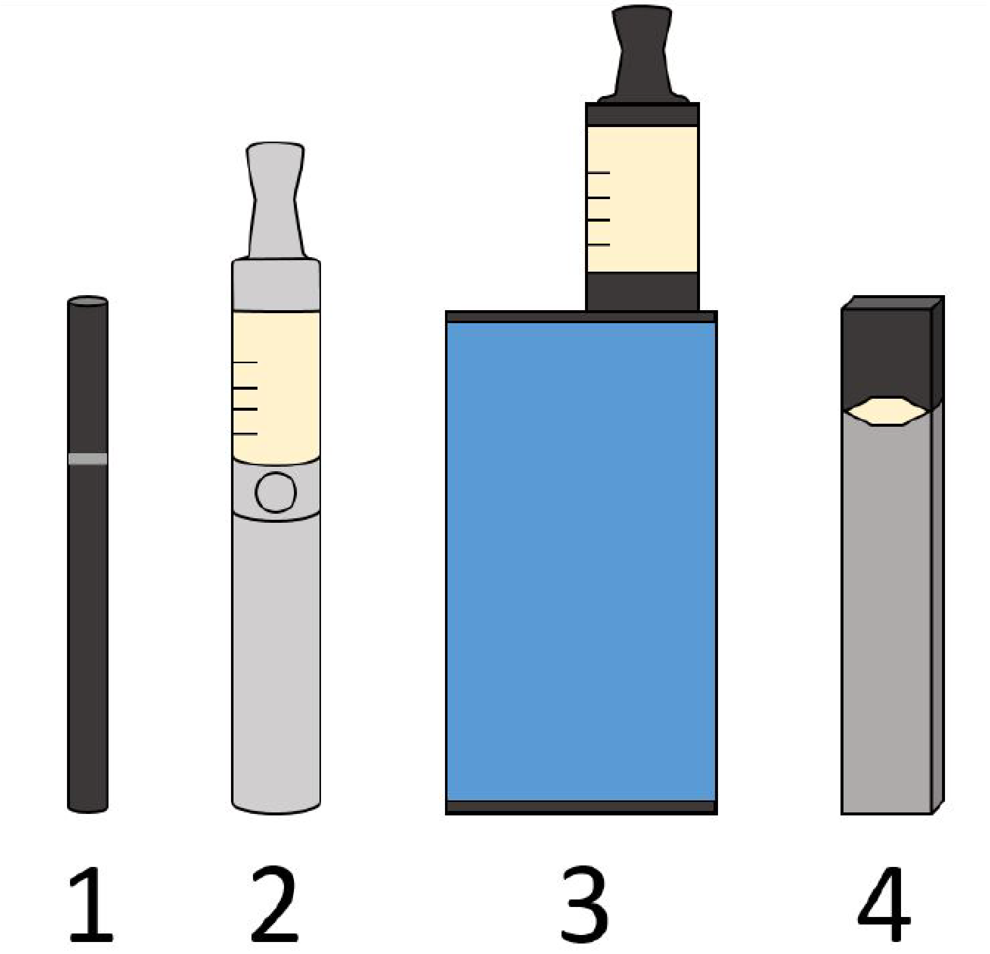

14. Which of the e-cigarette types in the above image do/did you use most regularly?

- ○ Device 1
- ○ Device 2
- ○ Device 3
- ○ Device 4

(Please choose option that applies best to you. If you no longer use e-cigarettes, please answer according to your past use.)

---

15. If you use more than one type of e-cigarette, which type do you use next most often?

- ○ Device 1
- ○ Device 2
- ○ Device 3
- ○ Device 4

(If you only use one type of e-cigarette please leave this question blank)

---

16. Is your most-used atomizer/cartomizer "low resistance" or "sub-ohm"?

- ○ Yes
- ○ No
- ○ Don't know

(If you no longer use e-cigarettes, please answer according to your past use.)

---

17. Do you adjust the settings (e.g. wattage) on your device to alter vapour production?

- ○ Yes
- ○ No
- ○ Don't know

(If you no longer use e-cigarettes, please answer according to your past use.)

---

18. How many models of e-cigarette did you use prior to the one you currently use now?

____

(Enter "0" if your current device is the only one you've ever used)

---

19. Where do you most commonly purchase your e-cigarettes and liquids?

- ○ Physical store in Australia
- ○ Physical store overseas
- ○ Online from Australian supplier
- ○ Online from overseas supplier
- ○ I don't know, I didn't buy it
- ○ I don't know

(If you no longer use e-cigarettes, please answer according to your past use.)

---

20. What flavour e-liquid do you use most often?

- ○ Tobacco
- ○ Fruit
- ○ Menthol/Mint
- ○ Candy
- ○ Dessert
- ○ Cereal
- ○ Drink/Beverage
- ○ Cinnamon
- ○ Unflavoured
- ○ Other

(Please choose option that applies best to you. If you no longer use e-cigarettes, please answer according to your past use.)

---

21. How many different flavours do you use on a regular basis?

- ○ 1
- ○ 2-3
- ○ 4-5
- ○ More than 5

(If you no longer use e-cigarettes, please answer according to your past use.)

---

22. Which statement best describes your use of different e-juice flavours?

- ○ I stick to only a few favourite flavours
- ○ I try new flavours occasionally but mainly use a few favourites
- ○ I am constantly trying new flavours

(If you no longer use e-cigarettes, please answer according to your past use.)

---

23. What is your preferred nicotine level?

- ○ 0 mg/mL (no nicotine)
- ○ Less than 6mg/mL (0.6%) but not nicotine free
- ○ 7 - 11 mg/mL (0.7 - 1.1%)
- ○ 12 - 19 mg/mL (1.2 - 1.9 %)
- ○ 20 mg/mL or more (over 2%)
- ○ Don't know

(If you no longer use e-cigarettes, please answer according to your past use.)

---

24. What is your preferred PG/VG ratio?

- ○ Equal (50PG/50VG)
- ○ High PG (eg. 70PG/30VG)
- ○ High VG (eg. 30PG/70VG)
- ○ Don't know
- ○ I don't care about the ratio

(If you no longer use e-cigarettes, please answer according to your past use.)

---

25. Do you mix your own e-liquids (e.g. add your own nicotine to e-juices or create your own flavours)?

- ○ Yes
- ○ No

(If you no longer use e-cigarettes, please answer according to your past use.)

---

26. Do you use nicotine salts?

- ○ Yes
- ○ No
- ○ Don't know

(If you no longer use e-cigarettes, please answer according to your past use.)

---

27. Do you experience cravings to use e-cigarettes?

- ○ Frequently
- ○ Sometimes
- ○ Never
- ○ Don't know

---

28. How do you use your e-cigarette?

- ○ In separate sessions, similar to smoking a cigarette
- ○ As needed throughout the day with no defined sessions
- ○ Mainly in defined sessions with some 'top up' hits as needed
- ○ Other

(Please choose option that applies best to you. If you no longer use e-cigarettes, please answer according to your past use.)

---

29. Do you use e-cigarettes for recreational purposes (eg. cloud chasing)?

- ○ Yes
- ○ No

(If you no longer use e-cigarettes, please answer according to your past use.)

---

30. Where do you use your e-cigarette?

☐ Inside at my home
☐ Outside at my home
☐ Inside in public places (bars, shopping centres, etc.)
☐ Outdoors in public places (parks, outdoor events etc.)
☐ Inside at my workplace
☐ Outside at my workplace
☐ Alone
☐ With others present
☐ In the car

(Please select all that apply. If you no longer use e-cigarettes, please answer according to your past use.)

---

31. Are you typically able to use your e-cigarette in places smoking is banned?

○ Yes
○ No
○ Don't know

(If you no longer use e-cigarettes, please answer according to your past use.)

This section asks you about your experiences and perceptions of e-cigarettes

32. Why did you start using e-cigarettes?

☐ To help quit smoking
☐ To reduce cigarette use
☐ Because of the novelty
☐ Because friends or family recommended them
☐ Because they looked fun to use
☐ Because of the flavours of e-juice available
☐ To save money
☐ Because of interest in the 'vaping' community
☐ To improve my health
☐ To reduce the impact of my smoking on others
☐ To be able to use them where I am not able to smoke cigarettes

(Please select all that apply)

---

33. Why did you stop using e-cigarettes?

☐ I just wanted to try it
☐ Wasn't the same as smoking tobacco cigarettes
☐ Didn't like the taste
☐ Too expensive
☐ Too difficult / messy to use
☐ Concerned about health risks
☐ Experienced unpleasant effects (eg. cough)
☐ Didn't help me quit/reduce smoking
☐ I successfully quit smoking and didn't need to use them anymore
☐ Other

Please list any other reasons why you stopped using e-cigarettes

____

---

34. Have you ever experienced any of the following due to using e-cigarettes?

☐ Cough
☐ Sore / irritated throat
☐ Nausea
☐ Dizziness
☐ Headache
☐ I have experienced other unpleasant effects
☐ I have not experienced any of these

(Please select all that apply)

---

35. Please list any other unpleasant effects you have experienced

____

---

36. Where do you go to find trusted information about e-cigarettes (recommendations, safety information etc.)?

☐ Online forums (e.g. E-Cigarette Forum, AussieVapers)
☐ Newspapers
☐ Television news reports
☐ Online news articles
☐ Scientific research papers
☐ Government publications (e.g. the Therepeutic Goods Administration)
☐ Social media (e.g. Reddit, Facebook, Instagram)
☐ Vape shops (online)
☐ Vape shops (physical store)
☐ Conversations with friends/family
☐ E-cigarette advertising material

(Please select all that apply)

---

37. Please tell us how strongly you agree with the following statements regarding e-cigarettes and traditional tobacco cigarettes:

	Strongly agree	Agree	Neutral	Disagree	Strongly disagree	Don’t know
E-cigarettes are completely safe to use long-term	○	○	○	○	○	○
E-cigarettes are significantly less harmful than tobacco cigarettes	○	○	○	○	○	○
E-cigarettes are just as harmful as tobacco cigarettes	○	○	○	○	○	○
E-cigarettes are more harmful than tobacco cigarettes	○	○	○	○	○	○

People who smoke tobacco cigarettes should switch to e-cigarettes to improve their health	○	○	○	○	○	○
People who have never smoked tobacco cigarettes should not use e-cigarettes	○	○	○	○	○	○
E-cigarettes are useful in helping people quit or reduce smoking	○	○	○	○	○	○
I would use a government-approved e-cigarette containing nicotine instead of my current device & juice	○	○	○	○	○	○
E-cigarettes are safe to use around other people	○	○	○	○	○	○
The government should allow e-cigarettes to be used in smoke-free areas	○	○	○	○	○	○
E-cigarettes should be banned until there is more evidence that they are safe	○	○	○	○	○	○
There is sufficient evidence that e-cigarettes are safe	○	○	○	○	○	○
E-cigarette should be regulated in the same way as traditional cigarettes and other tobacco products	○	○	○	○	○	○
E-cigarettes should be classified as a drug-based product (like other pharmaceutical nicotine replacement therapies, like patches and gum)	○	○	○	○	○	○
I am concerned by recent media reports of e-cigarette-related illnesses and deaths in the USA	○	○	○	○	○	○

38. What aspects of e-cigarettes would you like to learn more about?

____

(Please be specific)

39. Do you have any further comments about this survey?

____
